# A Minority of Athletes Pass Symmetry Criteria in a Series of Hop and Strength Tests Irrespective of Having an ACL Reconstructed Knee or Being Noninjured

**DOI:** 10.1177/19417381221097949

**Published:** 2022-06-27

**Authors:** Jonas L. Markström, Josefine E. Naili, Charlotte K. Häger

**Affiliations:** † Department of Community Medicine and Rehabilitation, Unit of Physiotherapy, Umeå University, Umeå, Sweden; ‡ Umeå School of Business, Economics and Statistics, Unit of Statistics, Umeå University, Umeå, Sweden; § Department of Women’s and Children’s Health, Karolinska Institutet, Stockholm, Sweden

**Keywords:** ACL reconstruction, hop testing, knee injury, rehabilitation, strength testing

## Abstract

**Background:**

Between-leg symmetry in 1-leg hop and knee strength performances is considered important after anterior cruciate ligament reconstruction (ACLR) to facilitate a safer return to sport. While few athletes with ACLR demonstrate symmetry in test batteries, reference data for noninjured athletes are lacking, thus questioning how ACLR-specific poor symmetry is.

**Hypothesis:**

Athletes with ACLR (hamstring autograft) show lower symmetry and have a lower proportion of symmetric individuals than noninjured athletes for knee flexion strength but not for hop for distance, vertical hop, and knee extension strength.

**Study design:**

Cross-sectional.

**Level of Evidence:**

Level 3.

**Methods:**

A total of 47 athletes with ACLR (median 13.0 months post-ACLR) who had returned to their sport, and 46 noninjured athletes participated. Symmetry was calculated between the worse and better legs for each test and combinations of them using the limb symmetry index (LSI_WORSE-BETTER_, ranging from 0% to 100%). The 2 groups were compared for these values and the proportions of individuals classified as symmetric (LSI_WORSE-BETTER_ ≥90%) using independent *t*-tests and Fisher’s exact tests, respectively.

**Results:**

Athletes with ACLR were less symmetric than noninjured athletes for knee flexion strength with a lower LSI_WORSE-BETTER_ (83% vs 91%, *P* < 0.01) and a lower proportion of symmetric individuals (39% vs 63%, *P* = 0.04). No differences between groups were revealed for the hop tests, knee extension strength, or combinations of tests (*P* > 0.05). Only 17% of the athletes with ACLR and 24% of the noninjured athletes demonstrated symmetric performances for all 4 tests.

**Conclusion:**

Athletes with ACLR (hamstring autograft) showed poorer symmetry in knee flexion strength than noninjured athletes, although both groups had few individuals who passed the test battery’s symmetry criteria.

**Clinical relevance:**

Symmetry is uncommon among athletes irrespective of ACLR and should be considered regarding expected rehabilitation outcomes and return-to-sport decisions post-ACLR.

Rupture of the anterior cruciate ligament (ACL) is a common and serious knee injury found mainly in sports,^25^ with acute and chronic physical and psychological consequences.^2,9,31^ Athletes are often treated surgically with ACL reconstruction (ACLR) and undergo physiotherapy-led rehabilitation programs for a successful return to sport. Despite these efforts, reports show that only 30% to 50% return to the same sport level within 12 to 24 months after ACLR.^3,4,10^ Unfortunately, about 1 in 5 persons younger than 25 years who return to sport suffer a reinjury on the ipsilateral or contralateral leg.^33^ Therefore, return-to-sport criteria aimed to decrease the risk of reinjury are continually discussed.^5,9,27,30,31^

A standard return-to-sport criterion after ACLR is a sufficient function of the injured leg, demonstrated with limb symmetry index (LSI, injured leg/healthy leg × 100) of at least 90% in a battery of hop and strength tests.^5,27,30^ Research reports a decreased risk of reinjury when achieving LSI ≥90% for knee extension strength,^13^ and different hop and strength tests included in test batteries both with and without patient-reported outcome measures.^12,18^ Unfortunately, a minority of ACL injured persons tested 6 to 24 months after ACLR demonstrate LSIs ≥90% for a battery of hop and strength tests, ranging between 0% to 29%,^6,7,28,29^ although occasionally higher with 57%.^32^ These proportions were often concluded to be alarmingly low and in urgent need of improvement.

However, none of these studies included a noninjured control group and, therefore, may have falsely attributed the observed low symmetry to the injury.^19^ In fact, previous research has reported strength asymmetries up to and exceeding 10% to be common among noninjured football and soccer athletes.^8,11^ Even elite sprinters, who presumably use their lower limbs equally when sprinting, have shown more than 15% asymmetry in lower limb muscle volume.^15^ Therefore, athletes should not be assumed to demonstrate symmetry in a battery of functional tests. Further research comparing symmetry in hop and strength outcomes between individuals with ACLR and noninjured control subjects is needed to inform expected results. Such information is necessary to better understand the evaluation of symmetry as a construct among individuals with ACLR.

This study aimed to evaluate if athletes with ACLR were equally symmetric as noninjured athletes in lower limb function by comparing the groups for LSI and the proportions of symmetric individuals (LSI ≥90%) in maximal single-leg hops for distance and height and knee extension and flexion strength. We hypothesized that athletes with ACLR would show a lower LSI and have a lower proportion of symmetric individuals than noninjured athletes for knee flexion strength but not for the other outcomes. We based this hypothesis on the fact that all participants had hamstring autografts and that muscle weakness postsurgery is dependent on the graft donor site.^35^

## Methods

### Participants

Participants were 47 athletes with ACLR and 46 noninjured athletes (CTRL) (Table 1). All participants were sports-active, in most cases in multiple sports, at a recreational or competitive level. Athletes in the ACLR group were recruited prospectively from the regional hospital’s orthopaedic clinic and, in a few cases, from a local sports medicine clinic and advertisements around the university and hospital campus. The inclusion criteria were: 17 to 34 years of age, unilateral ACL injury, returned to physical activity and feeling confident in performing maximal hop and strength tests, no concomitant injuries including a complete tear of any other knee ligament or major menisci or articular damage and no severe ankle sprain in the last 6 months or other musculoskeletal or neurological pathology that would affect test performance. All athletes in the ACLR group had an ipsilateral hamstring graft since this is standard practice nationally.^26^ Similar relevant criteria were applicable for the CTRL group, recruited from advertisements at the university and hospital campus, from sports clubs, and by word of mouth.

**Table 1.** Group characteristics

	ACLR N = 47	CTRL N = 46
Men:women, n	18:29	6:40
Age, years, mean (SD)	24.6 (4.7)	22.4 (3.3)
Months after surgery, median (Q1, Q3)	13.0 (10.5, 20.9)	N/A
Body height, m, mean (SD)	1.73 (0.08)	1.70 (0.07)
Body mass, kg, mean (SD)	71.4 (10.9)	64.9 (7.7)
BMI, kg/m^2^, mean (SD)	23.7 (2.5)	22.3 (2.0)
Sports played		
Soccer, n (%)	17 (36.2)	5 (10.9)
Floorball, n (%)	13 (27.7)	16 (34.8)
Winter sports, n (%)	5 (10.6)	1 (2.2)
Gymnastics/martial arts, n (%)	4 (8.5)	1 (2.2)
Multiple sports, n (%)	8 (17.0)	23 (50.0)

ACLR, athletes with anterior cruciate ligament reconstruction; BMI, body mass index; CTRL, noninjured athletes; Q1 and Q3, first and third quartiles; N/A, not applicable.

The test leader screened all participants for injury history through interviews via telephone before testing and then again at the time of testing. An experienced physiotherapist performed a clinical knee examination to screen for musculoskeletal injuries. After these screening procedures, participants were tested at the U-motion laboratory, Umeå, Sweden. Before partaking in the study, all participants provided written informed consent in agreement with the Declaration of Helsinki. The regional ethical review board in Umeå, Sweden, approved this study (Dnr. 2015/67-31).

### Test Procedure

#### Hop Testing

Participants performed 2 maximal hop tests to evaluate lower limb function; the 1-leg hop for distance (OLHD) and the 1-leg vertical hop (OLVH). These tests are highly able to discriminate between the leg with ACLR and the healthy contralateral leg,^14,16^ and show high test-retest reliability.^14,17^ The OLHD was performed first, followed by the OLVH. Both hop tests were performed barefoot and with arms behind the back, holding onto a 25-cm-long rope with knots on each side, similar to previous procedures.^14,17^ Participants had up to 2 practice trials to familiarize themselves with the hop tests before performing the test. Participants completed 3 to 5 hop trials per leg depending on whether the hop distance increased for each consecutive hop. A hop trial was classified as successful when the participant maintained a single leg stance for around 3 seconds after landing without putting the other foot down, shuffling around the standing foot to maintain stability, or letting go of the rope. The first hop trial was performed with the ACLR leg for athletes with ACLR and the nondominant leg (nonpreferred leg to kick a ball) for noninjured athletes. After that, both groups alternated between legs for each consecutive trial. Participants had rest of about 10 seconds between hop trials (thus ~20 s rest between trials on the same leg) and a rest of around 5 minutes rest after completing the OLHD before performing the OLVH.

For the OLHD, participants stood upright on 1 leg holding the rope behind their back, hopped forward as far as possible, and landed on the same leg. They were told to follow through forcefully when they initiated the hop (ie, not slowly bending the performing leg and then follow through). Participants were instructed to ‘stick’ the landing and regain control as quickly as possible.

For the OLVH, participants stood upright on 1 leg holding the rope behind their back, hopped as high as possible, and landed on the same leg. Similar to performing the OLHD, they were told to follow through forcefully when they initiated the hop and to ‘stick’ the landing and regain control as quickly as possible.

#### Isometric Knee Strength Testing

The isometric strength testing was performed in an isokinetic dynamometer (Kinetic communicator 125 Auto Positioning). Participants were seated in the dynamometer following the retailer’s recommended settings with a back angle of 78°, a seat bottom angle of 10°, and the knee at around 65° (0° defined by the lever arm in a horizontal position). The 65° knee angle was chosen for maximal isometric strength output for both knee extension and flexion^23^ and was similar to previous protocols.^12,36^ Participants were secured using straps around the hip, both shoulders, and the thigh being tested. The dynamometer axis was aligned with the lateral femoral epicondyle and with the lowest part of the resistance pad placed around 1 cm proximal to the medial malleolus. A zero-baseline correction was applied for each participant’s leg before data were collected. Participants had a warm-up of 2 trials of around 2 seconds each with submaximal contraction, with instructions to aim for an effort relative to the maximal effort of around 80% for the first trial and 90% for the second trial. After the warm up, 3 maximal 5-second trials were conducted, separated with a rest of around 5 seconds between repetitions. The healthy leg among individuals with ACLR and the dominant leg among controls were tested first for knee extension strength and then knee flexion strength, followed by testing of the other leg. For knee extension, participants were told to contract their quadriceps maximally by trying to extend their leg as forcefully as possible. For knee flexion, participants were told to contract their hamstrings maximally by trying to bend their leg as forcefully as possible.

### Data Analysis and Outcome Measures

The individuals included in this study were part of a larger project aiming to investigate the consequences of ACLR on movement patterns.^20-22^ Therefore, a motion capture system was used to evaluate hop performances. A total of 56 passive spherical markers were used to construct a 15-segment 6 degrees of freedom model. The test leader attached these markers with double-coated adhesive tape on the skin at anatomical landmarks, as previously described in detail.^20-22^ The marker coordinates were registered using a 3-dimensional motion capture system with 8 cameras (240 Hz, Oqus 300, Qualisys AB). The data were then exported to, and processed with, Visual3D software (v.5.02.19, C-Motion Inc.).

The OLHD length was calculated from the displacement of a marker on the testing leg’s foot between starting position to landing. The OLVH height was calculated from the displacement of the pelvis center of mass between standing to peak height. The maximal hop trial for each leg and test was extracted and analyzed. For knee extension and flexion strength, the dynamometer data were filtered with a moving average of 60 ms, and the single highest peak value was normalized to body mass and used in analyses. Note that 1 participant in the ACLR group did not perform strength testing since the dynamometer was unavailable at this particular testing.

Symmetries of the maximal hop and strength outcomes were evaluated with the LSI calculated between the worse and better leg for each test (LSI_WORSE-BETTER_, maximal value 100%). An individual was classified as symmetric for the maximal hop and strength outcomes when presenting an LSI_WORSE-BETTER_ of ≥90% (standard criterion for LSI_INJURED-HEALTHY_).^5,27,30^

The LSI_WORSE-BETTER_ has the advantage of evaluating the absolute value of symmetry without considering a prespecified between-leg comparison, which provides a better estimate of symmetry on a group level. For example, 3 individuals with LSIs calculated between the dominant and nondominant leg of 85%, 90%, and 120% result in a group mean value of 98% with a standard deviation of 15%. In contrast, the corresponding LSI_WORSE-BETTER_ of 85%, 90%, and 83% (LSI inverse of 120%) results in a group mean value of 86% with a standard deviation of 3%. The different LSI averages of 98% and 86% result in opposite conclusions in symmetry for these individuals when combined. The large differences in standard deviations of 15% versus 3% further motivate the LSI_WORSE-BETTER_ as the better estimate of symmetry when a prespecified between-leg comparison is not the main interest.

### Statistical Analyses

First, Pearson’s correlations were performed for the time between ACLR surgery and testing and the symmetry outcomes to evaluate possible associations before further analysis. However, there were no significant correlations (*P* = 0.28-0.87), meaning that symmetry seemed not to improve or deteriorate within these time-frames postsurgery. All athletes in the ACLR group were therefore analyzed as 1 group.

The aim to evaluate whether the ACLR group were as symmetric as the CTRL group was assessed by analyzing group averages in LSI_WORSE-BETTER_ in the hop and strength tests with independent *t*-tests (2-sided) and the proportions of symmetric individuals in these tests and for combinations of tests (both hop tests, both strength tests, all 4 tests) with Fisher’s exact tests (2-sided). Results for the *t*-tests were presented with effect sizes (Cohen’s *d* or, if different standard deviations between groups, Glass’s *delta*) classified with 0.2 for small, 0.5 for moderate, and ≥0.8 for large. The Statistical Package for the Social Sciences (v.25, IBM SPSS Statistics) was used with *P* < 0.05 determining statistical significance.

## Results

### Symmetry in Hop and Strength Performances

**Figure 1.**
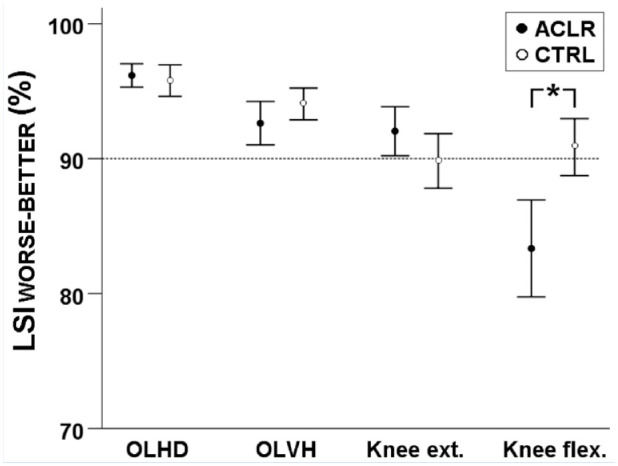
Group mean data with 95% CIs of LSI between the worse and the better leg (LSI_WORSE-BETTER_) for tests among athletes with ACLR and noninjured athletes. The dashed line indicates the standard 90% LSI cut-off. ACLR, athletes with anterior cruciate ligament reconstruction; CI, confidence interval; CTRL, noninjured athletes; Knee ext., knee extension strength; Knee flex., knee flexion strength; LSI, limb symmetry index; OLHD, 1-leg hop for distance; OLVH, 1-leg vertical hop. *Statistical between-group difference with *P* < 0.05.

The ACLR group demonstrated an 8% (95% confidence interval [CI], 3-12%) lower average LSI_WORSE-BETTER_ for knee flexion strength than the CTRL group (83% vs 91%, respectively, t[72.7] = −3.636 [corrected for a significant Levene’s test for inequality of variances]; *P* < 0.01; effect size *delta* = 1.07 [strong]) (Figure 1). However, the ACLR group did not show lower LSI_WORSE-BETTER_ than the CTRL group for OLHD (96% vs 96%, t[91] = 0.531; *P* = 0.60; effect size *d* = 0.12), OLVH (93% vs 94%, t[91] = −1.436;*P* = 0.15; effect size *d* = 0.31), or knee extension strength (92% vs. 90%, t[90] = 1.635; *P* = 0.11; effect size *d* = 0.34). Data for the hop and strength outcomes are presented in Table 2.

**Table 2.** Data for maximal hop and strength performances, presented as mean (SD)

	ACLR				CTRL			
	Injured	Healthy	Worse	Better	Non-dom.	Dom.	Worse	Better
OLHD, m	1.25 (0.21)	1.27 (0.21)	1.23 (0.21)	1.28 (0.21)	1.25 (0.22)	1.27 (0.23)	1.24 (0.23)	1.29 (0.22)
OLVH, m	0.24 (0.05)	0.25 (0.05)	0.23 (0.05)	0.25 (0.05)	0.23 (0.04)	0.24 (0.04)	0.23 (0.04)	0.24 (0.04)
Knee ext., Nm/kg	2.69 (0.64)	2.84 (0.62)	2.65 (0.60)	2.88 (0.64)	2.44 (0.50)	2.53 (0.59)	2.35 (0.53)	2.62 (0.55)
Knee flex., Nm/kg	1.07 (0.26)	1.24 (0.25)	1.05 (0.26)	1.26 (0.24)	1.12 (0.23)	1.16 (0.24)	1.08 (0.22)	1.19 (0.23)

ACLR, athletes with anterior cruciate ligament reconstruction; CTRL, noninjured athletes; Dom., dominant leg; Knee ext., knee extension strength; Knee flex., knee flexion strength; Non-dom, nondominant leg; OLHD, 1-leg hop for distance; OLVH, 1-leg vertical hop.

### Proportions of Symmetric Individuals in Hop and Strength Performances

**Figure 2.**
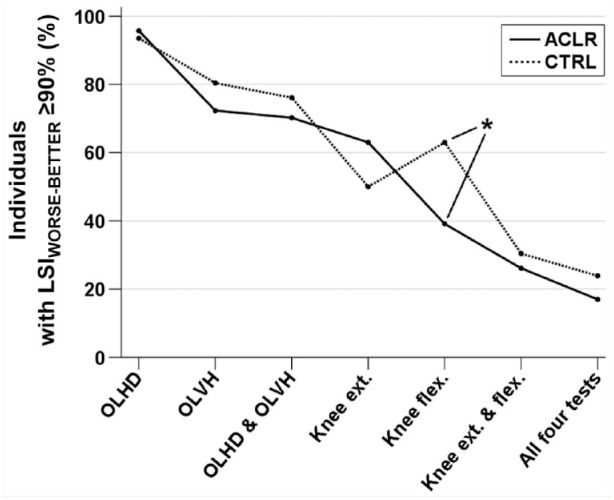
The proportion of individuals with an LSI between the worse and the better leg (LSI_WORSE-BETTER_) ≥90% for tests among athletes with ACLR and noninjured athletes. ACLR, athletes with anterior cruciate ligament reconstruction; CTRL, noninjured athletes; Knee ext., knee extension strength; Knee flex., knee flexion strength; LSI, limb symmetry index; OLHD, 1-leg hop for distance; OLVH, 1-leg vertical hop. *Statistical between-group difference with *P* < 0.05.

The ACLR group had a 24% lower proportion of individuals with LSI_WORSE-BETTER_ ≥90% for knee flexion strength than the CTRL group (39% vs 63%, respectively, *P* = 0.04) (Figure 2). However, the ACLR group did not show lower proportions of individuals with LSI_WORSE-BETTER_ ≥90% than the CTRL group for any of the remaining comparisons: OLHD (96% vs 93%, *P* = 0.49), OLVH (72% vs 80%, *P* = 0.25), OLHD and OLVH (70% vs 76%, *P* = 0.34), knee extension strength (63% vs 50%, *P* = 0.15), knee extension and flexion strength (26% vs 30%, *P* = 0.41), all tests (17% vs 24%, *P* = 0.30).

Further, 1 participant in each group (2%, respectively) demonstrated LSI_WORSE-BETTER_ <90% for both OLHD and OLVH, while 11 persons (24%) in the ACLR group and 8 persons (17%) in the CTRL group revealed LSI_WORSE-BETTER_ <90% for both knee extension and flexion strength. No individual in any of the groups had LSI_WORSE-BETTER_ <90% for all 4 tests.

## Discussion

The main finding of this study was that few athletes passed symmetry criteria in a battery of 2 hop and 2 strength tests independently of having had ACLR or being noninjured. Only 17% of athletes with ACLR that had returned to their sport and 24% of noninjured athletes demonstrated ≥90% symmetry in all 4 tests. The only difference between the groups was that the ACLR group showed poorer symmetry for knee flexion strength; they had an 8% lower average LSI_WORSE-BETTER_ for knee flexion strength, which was supported with a large effect size of 1.07, and a 24% lower proportion of individuals with an LSI_WORSE-BETTER_ ≥90% for knee flexion strength than the CTRL group. We expected this difference since all individuals in the ACLR group had a hamstring autograft, and muscle weakness after ACLR depends on the graft donor site.^35^

Evaluating LSIs for hop and strength performances is considered a valuable and straightforward tool to use during and after rehabilitation to assess progress for return-to-sport decisions.^5,27,30^ Their advantages include the relatively fast assessment in clinical practice while showing high test-retest reliability,^1,24^ and being clinically relevant since LSIs ≥90% seem to be associated with a reduced risk of reinjury.^12,13,18^ Interestingly, our results showed that only 1 in 5 persons in the ACLR group and 1 in 4 persons in the CTRL group were classified as symmetric after a battery of 2 hop and 2 strength tests. For the ACLR group, our finding is in line with previous results where 0% to 29% of individuals with ACLR show LSIs ≥90% for different batteries of hop and strength tests.^6,7,28,29^ The lack of research investigating the proportion of individuals showing symmetrical performances for a battery of tests among noninjured persons restricts the generalization of our findings for the CTRL group. Still, there is some evidence among noninjured controls for the hop for distance test and knee strength.

For the OLHD, Wren and colleagues^34^ report similar proportions of individuals with LSI ≥90% among male and female athletes with ACLR tested 5 to 12 months after surgery (63%) and noninjured controls (62%). For strength, Grace et al^11^ show that 62% and 61% of noninjured high-school male football athletes had >90% symmetry for knee extension and flexion strength, respectively. Croisier and colleagues^8^ report similar results, where 61% of noninjured professional male and female soccer players demonstrated >85% symmetry in knee flexion strength. Our results are similar to these findings, with 50% and 63% among the CTRL group showing LSI_WORSE-BETTER_ ≥90% for knee extension and flexion strength, respectively. Since the proportion of individuals classified as symmetric decreases with additional tests (Figure 2), it seems that our result of only 24% showing symmetric performances is a valid representation of noninjured athletes. In addition, the noninjured athletes demonstrated a mean LSI_WORSE-BETTER_ just below the 90% cut-off (more specifically: 89.8%) for knee extension strength, as seen in Figure 1. Therefore, athletes with or without ACLR should not be expected to display symmetry when performing a battery of hop and strength tests. This knowledge is important to consider by the clinician, patient, sports coach, and others involved when discussing expectations in symmetry during ACL rehabilitation and the return-to-sport decision.

Clinicians adopting the view that LSIs ≥90% for a battery of functional tests are required before recommending an athlete with ACLR to return to sport will probably need to test the athlete on multiple occasions, considering that symmetry is uncommon. However, multiple test sessions and training program modifications aimed to achieve symmetry may transform this goal into an artificial construct that decreases its predictive association to reinjury found in previous studies.^12,13,18^ Individuals may consciously or unconsciously adapt their training and testing performances of both legs to reach this goal, especially if related to a return-to-sport decision. Further research is required to investigate if associations between symmetry in strength and test batteries to lower reinjury rates are affected by the number of testing sessions with or without modified goal-directed training programs during the rehabilitation. Also, researchers that evaluate symmetry among individuals with ACLR should incorporate a control group or reference data to decrease the risk of false-positive results attributed to the injury.

When evaluating symmetry during ACL rehabilitation, current recommendations include testing the healthy leg for hop and strength performances shortly after the primary injury to attain a reference of the leg’s physical functioning to be used for later evaluations.^32^ This approach may provide a better reference of physical functioning to the injured leg over the course of rehabilitation since it avoids possible knee function deficits affecting the healthy leg after inactivity.^32^ Another recommendation is to adhere to stricter symmetry criteria before recommending a return to sport, including LSIs ≥90% for hop tests and LSIs ≥100% for strength for athletes with ACLR aiming to return to pivoting, contact, or competitive sport.^27^ Adhering to stricter criteria for strength by increasing the LSI_WORSE-BETTER_ to ≥95%, we found that only a single person in the ACLR group (~2%) and 2 persons in the CTRL group (~4%) passed the test battery. Therefore, stricter criteria may not be the answer to facilitate an improved return to sport, as only about 1 in 50 athletes pass such criteria irrespective of having had ACLR or being noninjured. In fact, adhering to less strict symmetry criteria for all 4 tests better distinguished between the ACLR and CTRL groups, with criteria of LSI_WORSE-BETTER_ ≥85% revealing that 35% of individuals in ACLR versus 61% in CTRL passed (*P* = 0.02) compared with LSI_WORSE-BETTER_ ≥90% where 17% in ACLR versus 24% in CTRL passed (*P* = 0.30). The relatively larger number of individuals who passed the test battery in the CTRL versus ACLR groups depended mainly on their knee strength, revealing symmetry values between 85% and 90%. The symmetry criteria of LSI_WORSE-BETTER_ ≥85% seems a more realistic criterion for a test battery than the more commonly used 90% cut-off for ACLR persons when considering reference data for noninjured athletes.

This study has significant limitations. The ACLRs in this study were hamstring autografts only. The choice to analyze maximal values may be less representative than using the average of multiple trials. On the other hand, maximal performances may highlight more apparent side-to-side differences that otherwise are filtered out by including poorer trials when calculating mean or median values. These participants performed different sports, thus resembling a wider sports-active population. There were different male-female ratios between the groups due to difficulties recruiting noninjured male athletes, but including sex as a covariate in the analyses did not affect the results (*P* = 0.21-0.85). Also, the specific rehabilitation for the ACLR group was not known, and we did not restrict testing to a specific time after ACLR surgery. All of these factors raise concerns for the generalizability of our results.

## Conclusion

Low proportions of athletes (17%) with ACLR that had returned to physical activity and 24% of noninjured athletes passed the ≥90% symmetry criteria in a test battery consisting of hop for distance, vertical hop, and isometric knee extension and flexion strength. Only for knee flexion strength did the athletes with ACLR (hamstring graft) demonstrate less symmetry and a lower proportion of symmetric individuals than the controls. The fact that most athletes, irrespective of having had ACLR or being noninjured, fail symmetry criteria in a test battery of hop and strength tests is important to consider by clinicians, patients, sports coaches, and others when discussing expected outcomes during ACL injury management, especially concerning return-to-sport decisions.
